# Omitting Chest Tubes in Robotic Mediastinal Surgery: A Prospective, Protocol-Driven Analysis of Safety and Recovery

**DOI:** 10.3390/jcm15166438

**Published:** 2026-08-20

**Authors:** Mehlika İşcan, Ömer Yavuz, Reyhan Ertan, Ali Yeginsu

**Affiliations:** Başakşehir Çam ve Sakura City Hospital, 34480 İstanbul, Türkiye; omeryavuz3853@gmail.com (Ö.Y.); rertanertan@gmail.com (R.E.); yeginsu@gmail.com (A.Y.)

**Keywords:** robotic surgical procedures, no-drain surgery, chest tubes, mediastinum, enhanced recovery after surgery, postoperative pain

## Abstract

**Background:** Routine chest tube placement remains common in robotic mediastinal surgery despite its association with postoperative pain. We evaluated the safety and feasibility of a protocol-driven no-drain strategy for robotic mediastinal lesion resection. **Methods:** This prospective study analyzed 39 consecutive procedures performed in 38 patients. A strict intraoperative safety protocol, including a submerged air leak test, was utilized to determine drain omission. Procedures were categorized into no-drain (*n* = 21) and drain (*n* = 18) groups based on protocol adherence. **Results:** The no-drain strategy was implemented in 53.8% of procedures, including complex cases such as thymomas and large lesions (up to 160 mm). The no-drain group was associated with lower median VAS pain scores at 12 h (2.0 vs. 5.0, *p* < 0.001) and shorter hospital length of stay (20.0 vs. 52.0 h, *p* < 0.001). No patient in the no-drain group required reintervention for residual pneumothorax. **Conclusions:** Omitting chest tubes using a standardized safety protocol is feasible and safe in appropriately selected patients. This strategy was associated with lower postoperative pain and a shorter hospital stay, supporting the feasibility of a short-stay recovery pathway after robotic mediastinal surgery.

## 1. Introduction

Mediastinal surgery has undergone a paradigm shift over the last two decades with the widespread adoption of robotic-assisted thoracic surgery (RATS). The high-definition three-dimensional visualization and precise maneuverability of robotic systems in confined spaces allow for the dissection of complex mediastinal structures with minimal tissue trauma [1,2]. The core philosophy of minimally invasive surgery (MIS) is to reduce surgical stress, minimize postoperative pain, and accelerate functional recovery. However, despite these technological advancements in surgical technique, traditional approaches regarding chest tube management have not evolved at the same pace.

Many surgeons continue to routinely place chest tubes after robotic thymectomy or mediastinal lesion resection due to concerns regarding potential hemothorax, chylothorax, or occult air leaks, particularly in extended resections where both pleural spaces may be breached [1,3]. Yet, the current literature suggests that the primary source of postoperative pain in thoracic surgery is often not the trocar incisions but the irritation of the parietal pleura and intercostal nerves caused by the drainage tube [4,5].

In recent years, “tubeless” or “no-drain” strategies have gained traction in pulmonary resection within the context of enhanced recovery after surgery (ERAS) protocols [6,7]. However, the adoption of this strategy in mediastinal surgery remains heterogeneous and non-standardized. Most existing studies are retrospective and subject to selection bias; typically, drains are omitted only in “simple” cases or small tumors, while routine drainage persists for complex cases [8]. There is a lack of a prospective, evidence-based algorithm in the literature that defines which patients can be safely managed without a drain based on physiological parameters rather than tumor size or histology.

The aim of this study was to prospectively evaluate the safety and efficacy of a “no-drain” strategy based on a strict, predefined intraoperative safety protocol in patients undergoing robotic mediastinal lesion resection and thymectomy. We hypothesized that in patients meeting predefined criteria—negative air leak test and complete hemostasis—chest tubes could be safely omitted across a range of tumor sizes and dissection extents, and we explored whether a no-drain strategy might be accompanied by lower postoperative pain and shorter length of stay.

## 2. Materials and Methods

### 2.1. Study Design and Patient Selection

This single-center, prospective observational cohort study was conducted between December 2024 and December 2025 and is reported in accordance with the STROBE (Strengthening the Reporting of Observational Studies in Epidemiology) statement (File S1) [9]. It was designed as a feasibility (protocol implementation) cohort: because drain omission was governed by intraoperative criteria, the no-drain and drain groups represent different intraoperative risk strata defined by the protocol rather than randomized arms, and all comparisons are therefore descriptive and associative. As a feasibility (pilot) study, no formal a priori sample-size calculation was performed; the cohort comprised all consecutive eligible patients treated during the 12-month enrollment period, and the study was not powered to exclude uncommon adverse events. The study protocol was approved by the Başakşehir Çam ve Sakura City Hospital Review Board (Approval No.: 2024-KAEK-11; Date: 16 December 2024) and was conducted in accordance with the Declaration of Helsinki. Written informed consent was obtained from all participants prior to surgery, explicitly detailing the potential for a “no-drain” approach if intraoperative safety criteria were met.

We enrolled consecutive patients aged > 18 years who underwent robotic-assisted surgery for mediastinal lesions (including cysts, thymomas, and neurogenic tumors) using the da Vinci Xi Surgical System (Intuitive Surgical, Sunnyvale, CA, USA). Predefined exclusion criteria included: (1) elective conversion to thoracotomy (excluding safety conversions), (2) active pleural infection or empyema, (3) requirement for concomitant anatomical lung resection, and (4) a history of dense pleural adhesions preventing robotic assessment. No patients were excluded based on these criteria during the study period; thus, the final cohort represents a consecutive series of all eligible robotic mediastinal procedures performed at our institution.

### 2.2. Surgical Technique

All procedures were performed under general anesthesia with double-lumen intubation for single-lung ventilation. Patient positioning was strictly dictated by the surgical approach and target pathology. For lateral robotic thymectomies and anterior mediastinal lesion resections, patients were placed in a 30-degree semi-supine position with the ipsilateral side elevated. In contrast, for subxiphoid thymectomies, patients were positioned in a supine position. For middle and posterior mediastinal lesion resections, a full lateral decubitus position was utilized to maximize exposure of the posterior compartments. A standard three-port or four-port robotic configuration was employed depending on the specific approach selected (left-sided, right-sided, or subxiphoid).

Carbon dioxide (CO_2_) insufflation was maintained at a pressure of 8 mmHg to facilitate exposure. Dissection was performed using Cadiere forceps in the left hand and Maryland bipolar forceps in the right hand. This combination allowed for precise dissection and hemostasis without the need for additional thermal energy devices in all cases. All specimens were retrieved using an endoscopic bag to prevent port-site seeding.

### 2.3. The “Safety-First” Intraoperative Protocol for Drain Omission

**Figure 1:**
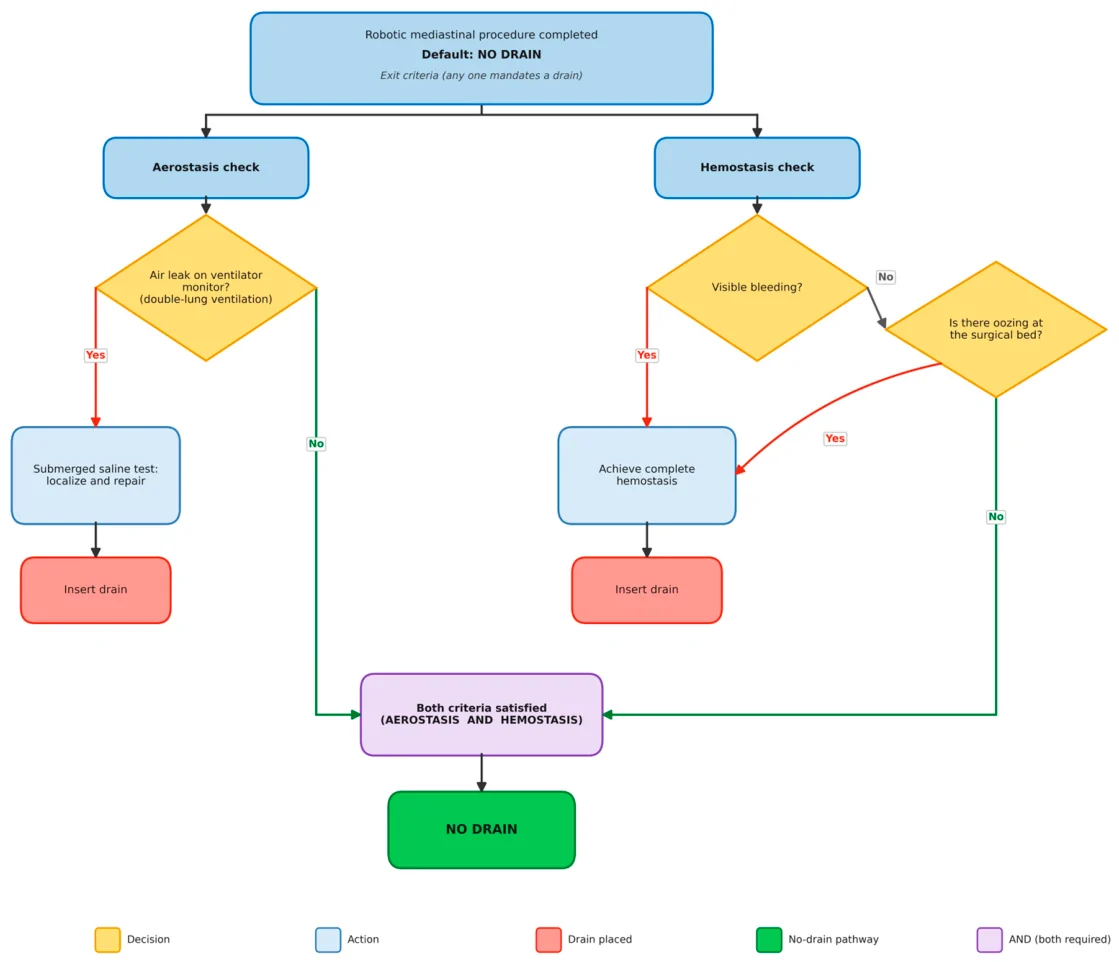
Safety-first intraoperative decision algorithm for chest tube placement versus omission. The default strategy is no drainage; two equally weighted exit criteria are evaluated independently—an aerostasis check and a hemostasis check. Aerostasis: if an air leak is seen on the ventilator monitor during double-lung ventilation, a submerged saline test is used to localize and repair the leak, and a chest tube is placed; if no leak is present, the criterion is satisfied. Hemostasis: if visible bleeding is present or if oozing is detected at the surgical bed, complete hemostasis is achieved, and a chest tube is placed; a completely dry field satisfies the criterion. When both criteria are satisfied, the no-drain pathway is followed; otherwise, a chest tube is placed. When placed, a 24F chest tube is managed on water-seal drainage and removed once the lung is fully expanded, no air leak is present, and drainage is <200 mL/day.

The decision to place a chest tube was governed by a strict, predefined intraoperative safety checklist, with a no-drain strategy as the default. Drain placement was based on two equally weighted exit criteria: an aerostasis check and a hemostasis check. When both criteria were satisfied, no chest tube was placed; otherwise, a chest tube was placed. The complete decision algorithm is summarized in Figure 1.

*Aerostasis check.* During double-lung ventilation, the ventilator monitor was inspected for an air leak. If no leak was present, the criterion was satisfied. If a leak was detected, a submerged saline test—filling the thoracic cavity with warm saline while the lung was inflated to a sustained 20–30 cmH_2_O—was used to localize and repair the leak, and a chest tube was placed.

*Hemostasis check.* The surgical bed was inspected under normal systemic blood pressure. If visible bleeding was present, complete hemostasis was achieved, and a chest tube was placed. If no visible bleeding was present, the bed was reassessed for oozing; if oozing was present, complete hemostasis was achieved, and a chest tube was placed, whereas a completely dry field satisfied the criterion.

When both criteria were satisfied, no chest tube was placed. When a chest tube was placed, a 24F tube was used. It was managed on water-seal drainage and removed once the lung was fully expanded, no air leak was present, and daily drainage was <200 mL/day.

### 2.4. No-Drain Closure Technique

For patients eligible for the no-drain approach, a suction catheter (16F) (Bıçakçılar; City-Country: İstanbul, Türkiye) was inserted through the camera port into the apical thoracic cavity. While the anesthesiologist performed a sustained Valsalva maneuver to fully re-expand the lung, the catheter was withdrawn under suction, and the port site was immediately sutured airtight.

### 2.5. Postoperative Management and Outcome Measures

All patients received the same standardized, opioid-sparing analgesia protocol, titrated to a 0–10 visual analog scale (VAS) [10]. Patients with mild pain (VAS ≤ 4) received scheduled intravenous paracetamol 1 g twice daily. When the VAS score exceeded 4 (i.e., ≥5, corresponding to the conventional threshold for moderate-to-severe pain requiring step-up analgesia [11]), paracetamol was increased to 1 g four times daily, and tramadol (50 mg twice daily) was added as rescue analgesia. No regional or local anesthetic technique (epidural, paravertebral, intercostal, or wound infiltration) was used in any patient at any stage, and no strong opioids were administered. Chest X-rays were obtained routinely at the 2nd and 12th postoperative hours and were reviewed and interpreted by the attending thoracic surgeons to rule out pneumothorax or significant effusion. If the patient remained hospitalized beyond 24 h, daily chest X-rays were performed until discharge. Discharge criteria were standardized and applied uniformly to both groups, consistent with enhanced recovery (ERAS) recommendations for thoracic surgery [12] and with published discharge criteria after thoracoscopic [13] and no-drain [14] resection. A patient was considered eligible for discharge when all of the following were met: adequate pain control on oral analgesia or no analgesic requirement; room-air oxygen saturation ≥ 95% with no supplemental oxygen requirement and no dyspnea; full lung expansion on chest radiography; removal of the chest tube or its prior absence—that is, no dependence on a drain; independent ambulation with the ability to perform self-care; tolerance of oral intake without nausea or vomiting; stable vital signs without fever or any active complication requiring inpatient care; and return of urinary and bowel function (micturition and passage of flatus or stool).

All patients were followed prospectively after discharge with scheduled in-person outpatient visits: a routine first visit at approximately 12 days and a second visit at approximately 30 days, when the final histopathology was reviewed. Postoperative complications, readmissions, and reinterventions within 30 days were ascertained at these visits and from the institutional electronic medical record. Thirty-day follow-up was complete for all patients (38/38, 100%).

Primary Endpoints: The primary efficacy endpoints were early postoperative pain and length of stay (LOS). Pain was assessed by an attending thoracic surgeon using a standardized 0–10 visual analog scale (VAS) presented to the patient at a single 12 h time point. This early time point lies within the first postoperative day, when acute surgical pain is most intense and clinically most relevant [15], while being early enough to capture all patients, including those discharged within 24 h; by this point, patients were also beyond the immediate anesthetic recovery period, so ratings reflected surgical rather than residual anesthetic effects. The primary safety endpoint was failure of the no-drain strategy, defined a priori as a composite of any of the following: postoperative chest tube insertion, reoperation, clinically significant pneumothorax or pleural effusion, readmission within 30 days, or mortality.

Secondary Endpoints: Secondary endpoints included the incidence of postoperative complications within 30 days, operative times, and exploratory patterns of drain use according to lesion size and pathology.

### 2.6. Statistical Analysis

Data analysis was performed using SPSS version 26.0 (IBM Corp., Armonk, NY, USA). Continuous variables were assessed for normality using the Shapiro–Wilk test and visual inspection of histograms. Normally distributed variables (e.g., age, BMI) were summarized as mean ± standard deviation (SD) and compared between groups using the independent-samples Student’s *t*-test; homogeneity of variances was assessed with Levene’s test. Non-normally distributed variables (e.g., VAS scores, operative time, length of hospital stay, and tumor size) were reported as median (interquartile range [IQR]) and compared using the Mann–Whitney U test. Categorical variables (e.g., gender, complication rates, pathology groups) were presented as *n* (%) and compared using Fisher’s exact test (or the Fisher–Freeman–Halton test for r × c tables, as appropriate) due to the limited sample size and small expected cell counts. For data visualization, scatter plots with jittering were used to reduce data overlap for ordinal variables (VAS scores). In addition, an exploratory bubble chart visualization was constructed to depict the relationship among operative time, tumor size, drain usage, and pain severity; no inferential regression modeling was performed for this visualization. All tests were two-sided, and a *p*-value < 0.05 was considered statistically significant. Between-group differences in continuous variables were summarized as Hodges–Lehmann median differences with 95% confidence intervals (CIs); exact (Clopper–Pearson) 95% CIs were reported for proportions.

## 3. Results

### 3.1. Baseline Characteristics and Patient Demographics

A total of 39 consecutive procedures were performed in 38 patients during the study period. There were no missing data for the analyzed variables. Following the strict intraoperative safety protocol, the “no-drain” strategy was implemented in 21 procedures (53.8%; 95% CI 37.2–69.9%), while 18 procedures (46.2%) required chest tube placement based on exit criteria. The two groups did not differ significantly in baseline demographic or preoperative clinical characteristics (Table 1): there were no statistically significant differences in age, gender, BMI, or ASA scores (*p* > 0.05), and the prevalence of comorbidities such as myasthenia gravis (14.3% vs. 33.3%, *p* = 0.255), history of smoking (52.4% vs. 22.2%, *p* = 0.098), and antiplatelet use (*p* > 0.99) was similar between the no-drain and drain groups. These comparisons concern preoperative variables only; because drain placement was determined by intraoperative findings, the two groups differ by design on these intraoperative determinants, which are not captured in Table 1. The absence of differences in baseline characteristics should therefore not be interpreted as indicating that the groups were otherwise comparable.

### 3.2. Perioperative Outcomes and Safety Profile

Intraoperative outcomes are detailed in Table 2. The median total operation time and console time were shorter in the no-drain group compared to the drain group (90 vs. 165 min, *p* = 0.076; 75 vs. 125 min, *p* = 0.067), although neither difference reached statistical significance.

Regarding safety, drain placement was governed by the predefined intraoperative exit criteria. One patient (5.6%) required tracheal repair during the dissection of a bronchogenic cyst adherent to the membranous tracheal wall. Although robotic primary repair was technically feasible, a loss of secure airway control precluded safe robotic completion. Consequently, an emergent conversion to thoracotomy was performed to secure the airway and complete the repair, necessitating subsequent drain placement. Thus, the patient was excluded from the no-drain pathway strictly per protocol. No intraoperative complications or conversions were observed in the patients who successfully met the no-drain criteria.

### 3.3. Postoperative Pain and Recovery

**Figure 2:**
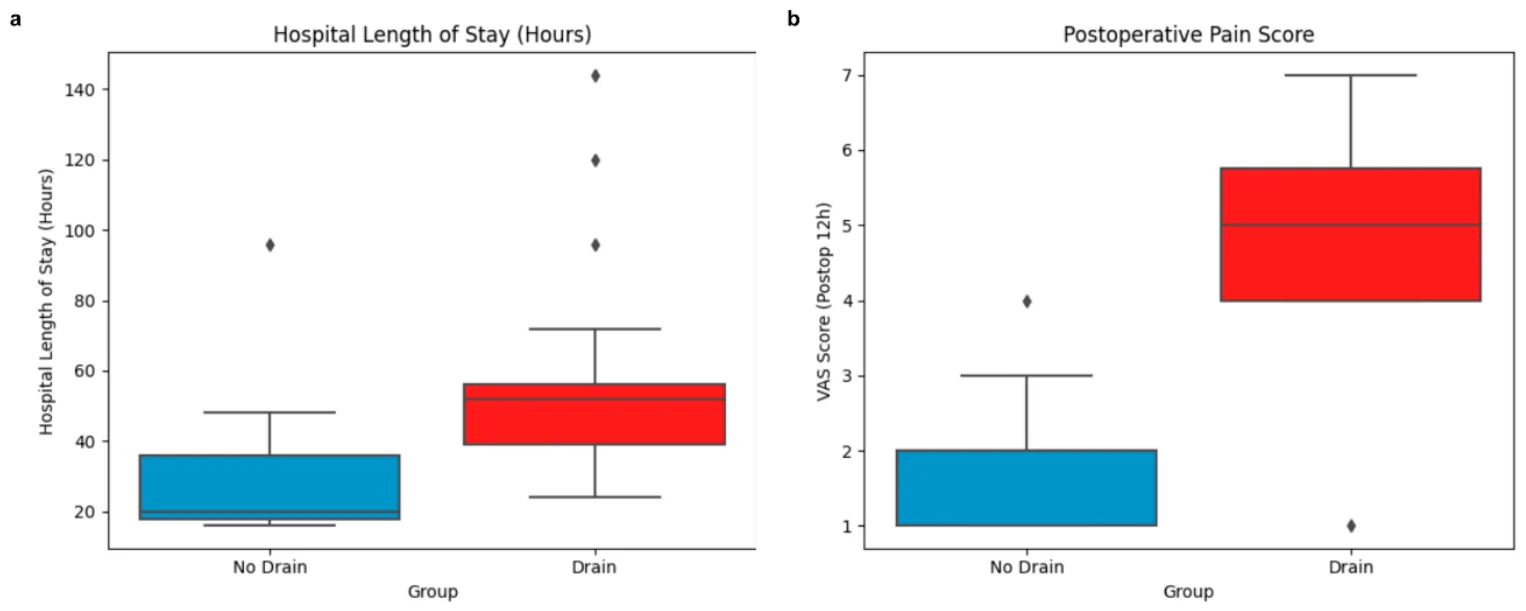
Primary postoperative outcomes in the no-drain and drain groups. (**a**) Hospital Length of Stay: Comparison of hospitalization duration between the no-drain (blue) and drain (red) groups. The median length of stay was shorter in the no-drain group (20 h vs. 52 h, *p* < 0.001), with discharge within the first 24 h in the majority of patients. (**b**) Postoperative Pain Score: Boxplot illustrating early postoperative pain levels (visual analog scale at 12 h). The median VAS score was lower in the no-drain group than in the drainage group (2.0 vs. 5.0, *p* < 0.001), indicating lower early postoperative pain in the no-drain group. Note: Box boundaries represent the interquartile range (IQR), the horizontal line inside the box indicates the median, and whiskers represent the range excluding outliers and diamonds (rhombuses) indicate individual outlier values.

Early postoperative pain scores and length of stay were lower in the no-drain group than in the drain group (Table 2). The median VAS pain score at 12 h post-surgery was lower in the no-drain group compared to the drain group (2.0 [IQR: 1.0–2.0] vs. 5.0 [IQR: 4.0–5.8]; median difference 3.0, 95% CI 2.0–4.0; *p* < 0.001). Consistent with these pain scores, escalation to four-times-daily paracetamol with rescue tramadol was required in no procedures of the no-drain group (0/21) versus 66.7% (12/18) of the drain group; no patient in either group required strong opioids. In the drain group, the median chest tube duration was 24.0 h (IQR: 24.0–48.0). The median LOS was also shorter in the no-drain cohort (20.0 h vs. 52.0 h; median difference 24.0 h, 95% CI 12.0–36.0; *p* < 0.001). These findings are visually corroborated by the boxplots in Figure 2, which demonstrate the distinct separation in pain levels and hospitalization duration between the groups.

### 3.4. Postoperative Complications

There was no 30-day mortality in either group. Postoperative complications were graded according to the Clavien–Dindo classification (Table 2). The overall complication rate was numerically lower in the no-drain group compared to the drain group (9.5% vs. 16.7%, *p* = 0.647).

In the no-drain group, complications were limited to two cases (9.5%) of minimal residual pneumothorax (Clavien–Dindo Grade I). Both patients remained asymptomatic and were managed conservatively with oxygen therapy and respiratory physiotherapy. Full lung expansion was confirmed radiologically within 72 h, and neither patient required tube thoracostomy or secondary invasive intervention. No failure of the no-drain strategy—defined as tube thoracostomy, reoperation, clinically significant pneumothorax or effusion, readmission, or mortality—occurred among the 21 no-drain procedures (0/21; exact 95% CI, 0–16.1%).

In contrast, the drain group experienced more severe complications involving three patients (16.7%). One patient developed a superficial surgical site infection at the drain insertion site (Grade II), which resolved with antibiotic therapy. Two patients (11.1%) required reintervention: one patient required bedside insertion of a small-bore catheter for symptomatic pleural effusion (Grade IIIa), and another underwent video-assisted thoracoscopic surgery (VATS) exploration (Grade IIIb) for suspected active bleeding (subsequently diagnosed as hemolytic anemia). Consequently, the reintervention rate was 11.1% in the drain group versus 0.0% in the no-drain group (*p* = 0.206).

### 3.5. Exploratory Visualization of Surgical Complexity and Clinical Outcomes

**Figure 3:**
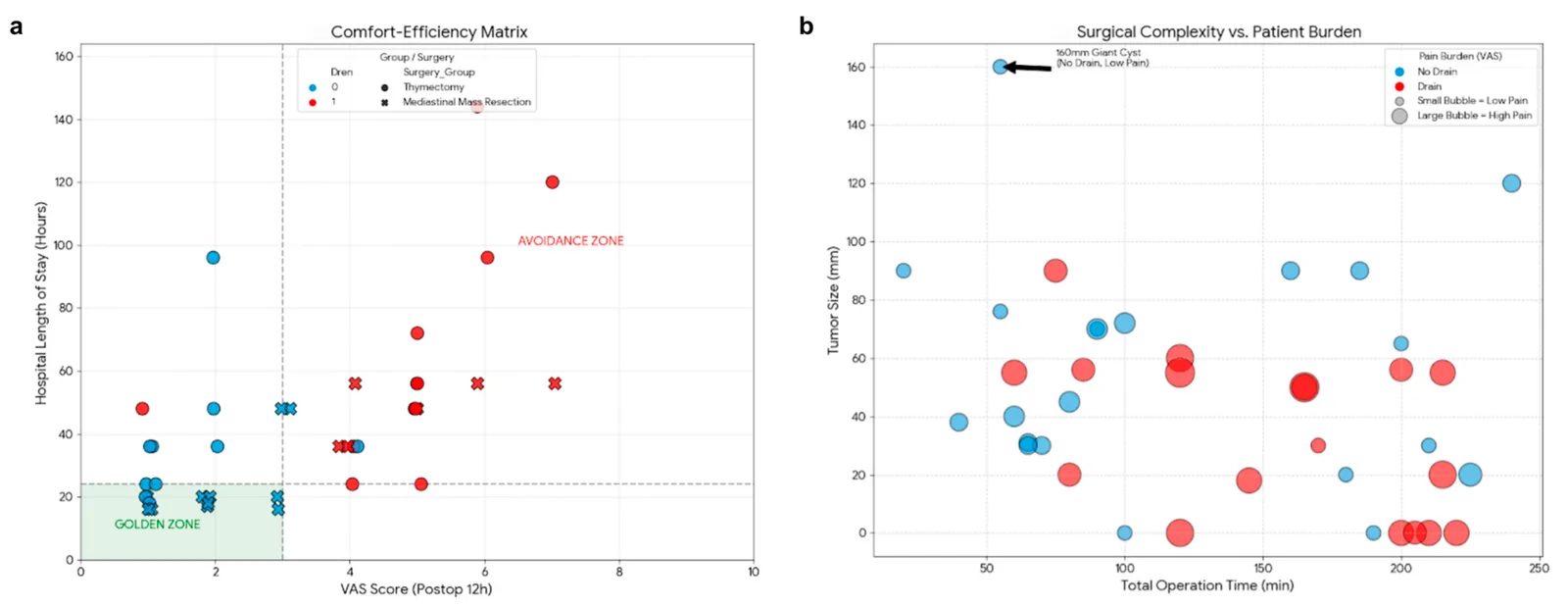
Exploratory visualization of clinical outcomes and surgical complexity. (**a**) The comfort–efficiency matrix: Scatter plot showing the relationship between early postoperative pain (VAS score at 12 h) and hospital length of stay. The no-drain cohort (blue dots) is predominantly clustered within the target “Golden Zone” (VAS ≤ 3 and Stay ≤ 24 h), corresponding to lower pain and a shorter stay. In contrast, the drainage group (red dots) is scattered towards the “Avoidance Zone,” characterized by higher pain scores and prolonged hospitalization. (**b**) Surgical Complexity vs. Patient Burden: A bubble chart showing tumor size (*y*-axis), total operation time (*x*-axis), and pain (bubble size). The largest lesion in the series (a 160 mm pulmonary sequestration with a giant cystic appearance, indicated by the arrow) was managed without a drain and resulted in minimal pain (small blue bubble). Conversely, in this cohort, some patients with a drain reported higher pain scores despite smaller tumor sizes or shorter operation times. These are descriptive, hypothesis-generating observations and do not establish a causal relationship.

Figure 3 presents exploratory visualizations of early pain and length of stay in relation to surgical complexity (tumor size and operative time). The comfort–efficiency matrix (Figure 3a) illustrates that the majority of patients in the no-drain group clustered within the targeted “Golden Zone” (VAS ≤ 3 and LOS ≤ 24 h). Furthermore, the bubble chart visualization (Figure 3b) illustrates the distribution of pain scores in relation to drain status, tumor size, and operative time in this cohort. The largest lesion in the series—a 160 mm posterior mediastinal lesion, radiologically cystic but pathologically confirmed as a pulmonary sequestration—was successfully managed without a drain, resulting in minimal pain (VAS 1) and early discharge.

### 3.6. Drain Usage According to Pathology

**Figure 4:**
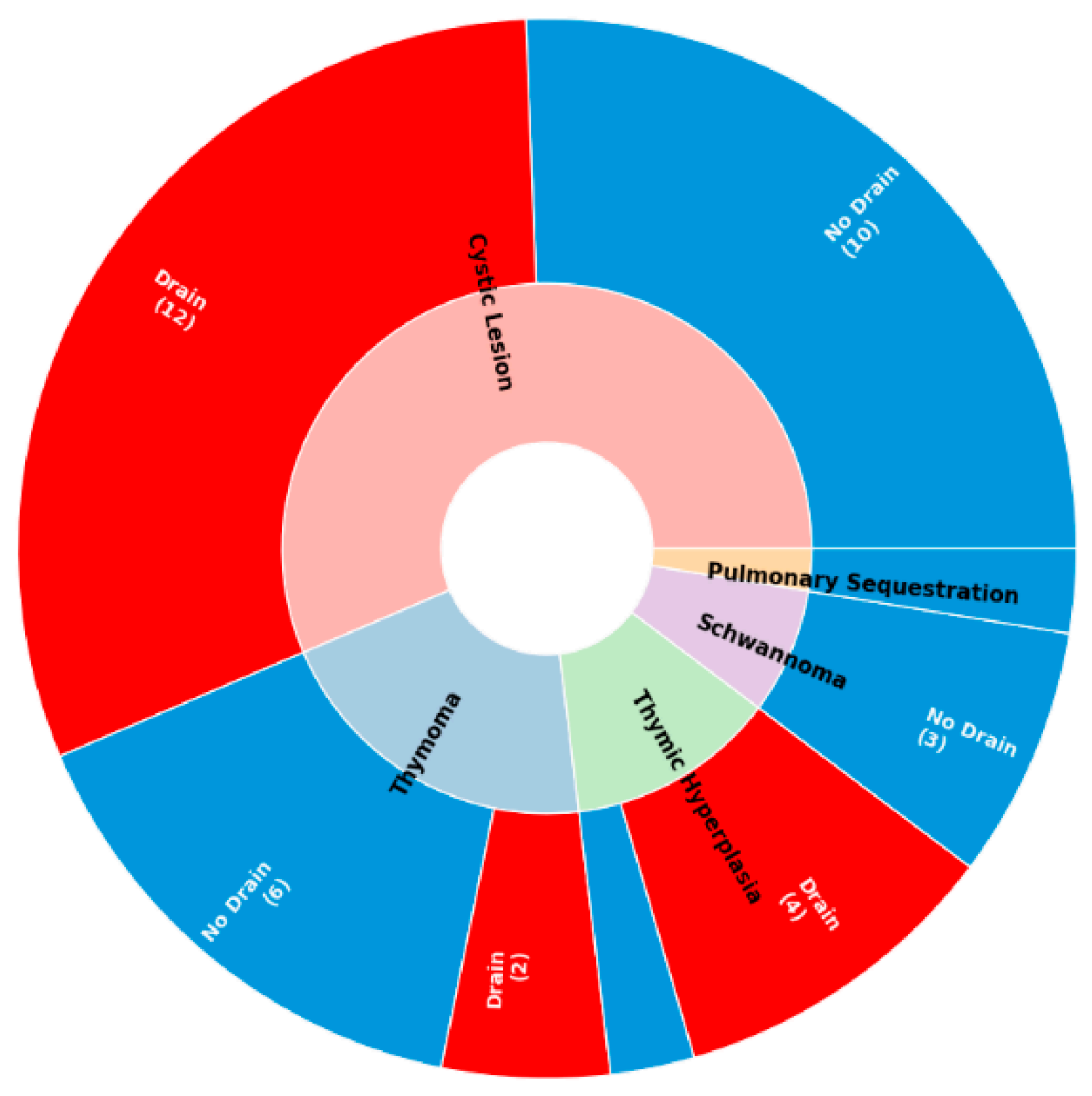
Hierarchical distribution of drain usage according to pathological diagnosis. The inner ring represents the pathological categories (slice width proportional to case volume), while the outer ring displays the drain status within each category (blue: no-drain, red: drain placed). Numbers in parentheses indicate the number of procedures in each segment. Key findings: (1) Thymoma: Contrary to the convention for solid tumors, 75% of thymoma procedures (6/8) were successfully managed without a chest tube (predominantly blue outer arc). (2) Cystic Lesions: Representing the largest cohort, cysts showed a balanced approach, with drain placement often dictated by adhesion severity rather than pathology alone. (3) Neurogenic/Rare Tumors: All schwannoma and pulmonary sequestration cases (100%) were discharged without a drain, highlighting the feasibility of the no-drain approach in posterior mediastinal surgery.

The distribution of drain placement varied by surgical procedure and pathological diagnosis (Figure 4 and Table 3). The no-drain rate was high for thymomas (75.0%, 6/8). This subgroup includes a particularly instructive case illustrating the feasibility of the no-drain approach even in reoperative settings: one patient underwent two separate robotic procedures—an initial resection of a cystic thymoma followed by a completion thymectomy—and both operations were successfully managed without a chest tube.

Conversely, patients with thymic hyperplasia had the lowest no-drain rate (20.0%, 1/5), reflecting the diffuse nature of the dissection and a lower threshold for drainage in myasthenia gravis cases. Regarding mediastinal lesion resections, neurogenic tumors (schwannomas, *n* = 3) and a posterior mediastinal pulmonary sequestration (*n* = 1) were managed entirely without drains (100%). In cystic lesions (thymic, bronchogenic, mesothelial, etc.), which constituted the largest subgroup across both surgical categories, drain usage was balanced (45.5% overall no-drain rate), often dictated by the extent of adhesions and the surface area of pleural dissection rather than the pathology itself.

## 4. Discussion

The primary finding of this prospective study is that a protocol-driven no-drain strategy in robotic mediastinal surgery is feasible and safe in appropriately selected patients and was associated with lower early postoperative pain scores and shorter hospital stays in this cohort. By adhering to strict intraoperative safety criteria, we successfully omitted chest tubes in 53.8% of procedures. This cohort was not limited to “simple” cysts; it included complex solid neoplasms such as thymomas (75% no-drain rate) and large lesions, including a 160 mm giant cyst-mimicking pulmonary sequestration. The no-drain strategy was associated with lower early postoperative pain (median VAS 2.0 vs. 5.0, *p* < 0.001) and a shorter hospital stay. Discharge within the first 24 h was observed in the no-drain group, with some patients discharged as early as 16 h postoperatively, supporting the feasibility of a short-stay recovery pathway after robotic mediastinal surgery. Historically, the dogma of routine drainage in thoracic surgery has been predicated on the fear of unrecognized hemorrhage or air leak [3]. However, the physiological cost of this practice is high. As demonstrated by Refai et al. [4] and Wildgaard et al. [5], the chest tube itself acts as a rigid nociceptive stimulus, irritating the periosteum and intercostal nerves with every respiratory excursion. These findings are consistent with this proposed mechanism. In the exploratory bubble chart (Figure 3b), higher pain scores were more frequently observed in the drain group—including in some patients with small lesions—than in patients with larger lesions managed without a drain. These are exploratory observations, however, and cannot distinguish an effect of drainage from confounding by indication, as drains were placed in patients with specific intraoperative findings. Because analgesia was identical and opioid-sparing in both groups, with no regional technique used, the between-group difference in pain is unlikely to reflect analgesic management; no no-drain patient required rescue tramadol, whereas two-thirds of the drain group did (0/21 vs. 12/18).

While several retrospective series have reported on no-drain VATS thymectomy, selection bias remains a major limitation in these studies. Drains are often omitted only in “ideal” candidates with small, non-invasive tumors [16,17]. Our study differentiates itself by applying a prospective default no-drain strategy that does not exclude patients based on tumor size. The successful management of a 160 mm cyst-mimicking pulmonary sequestration and multiple solid thymomas without drainage challenges the conventional wisdom that extensive dissection requires postoperative drainage. This supports the findings of Lu et al. [18], who suggested that even bilateral pleural opening during extended thymectomy is not an absolute contraindication to a no-drain approach, provided that air evacuation is meticulously performed.

Safety remains a central concern in adopting this strategy. Our protocol was designed with distinct “exit criteria”—an aerostasis check and a hemostasis check—to identify patients in whom a drain was indicated. In the single conversion case—a loss of secure airway control during dissection of a cyst adherent to the trachea—a drain was placed as mandated by the protocol, prioritizing patient safety over the no-drain goal. This case illustrates that the protocol’s default of no drainage is abandoned whenever intraoperative safety requires it, rather than being applied rigidly; it is presented as an example of the protocol in operation rather than as evidence of its efficacy. Although two patients in the no-drain group developed residual pneumothorax, neither required tube thoracostomy or surgical reintervention, and both resolved spontaneously with conservative management, suggesting that small residual air pockets were well tolerated and did not necessitate routine prophylactic drainage. The reintervention rate in the drain group was 11.1% (one VATS exploration and one catheter insertion).

Our findings resonate with the recent experience reported by McCormack et al. [19], who advocated for the complete elimination of routine chest tubes after reporting a 98.7% no-drain rate in robotic thymectomy. However, a fundamental distinction lies in the scope of the study populations: while their series was exclusively focused on thymectomies, our cohort encompassed the full spectrum of anterior and posterior mediastinal pathologies, including bronchogenic cysts, schwannomas, and complex thymic neoplasms. This broader applicability demonstrates that the no-drain strategy is not limited to thymic surgery but is feasible across diverse mediastinal etiologies. Furthermore, our subgroup analysis confirms the versatility of this protocol, achieving a 100% no-drain rate in neurogenic tumors and 75% in thymomas, challenging the hesitation often associated with solid tumor resections. Unlike a retrospective observation of a “default no-drain” policy, our prospective algorithm provides objective intraoperative criteria—such as the submerged air leak test—ensuring that the decision to omit the drain is based on reproducible physiological parameters. This offers a safer, standardized roadmap for centers adopting this technique across the entire spectrum of mediastinal surgery.

A central question is whether this protocol can be transferred to institutions with lower robotic volume or different perioperative pathways. Two features favor transferability. First, the decision to omit a drain rests on objective, reproducible intraoperative endpoints—a ventilator-monitored and submerged air leak test and a structured hemostasis check—rather than on operator experience; the value of such explicit, standardized criteria is underscored by the multi-institutional Guangzhou expert consensus on tubeless thoracoscopic surgery, which sought to codify patient selection and intraoperative requirements so that drain omission can be reproduced across centers [20]. Second, the algorithm is procedure-agnostic and is therefore not tied to a single high-volume indication. Nevertheless, three caveats temper broad generalization. (i) Achieving the strictly dry, airtight field that the protocol requires is more readily accomplished with the dexterity and magnified visualization of a robotic platform and with adequate experience; robotic thoracic proficiency follows a measurable learning curve, with the steepest gains over the first 15–20 thymectomy cases [21], so a lower threshold for drainage and closer early monitoring would be prudent during a program’s initial phase. (ii) The early-discharge benefit is not attributable to drain omission alone but also depends on perioperative infrastructure—standardized multimodal analgesia, same- or next-day radiographic follow-up, and outpatient pathways—whose implementation and impact vary substantially between institutions [22]. (iii) Our findings derive from a single experienced center. We therefore regard this protocol as a safe and reproducible framework in appropriately selected patients at experienced robotic centers, which warrants multicenter prospective validation across a range of institutional volumes and perioperative pathways before broader generalization.

The reduction in LOS has implications for healthcare resource utilization. The median LOS of 20 h in the no-drain group aligns with modern ERAS principles [12,23] and supports the feasibility of a short-stay, accelerated-recovery pathway in robotic thoracic surgery. Omitting the drain also removes a physical restriction on mobility and reduces the nursing workload associated with drain care. Together with lower pain scores, this may contribute to the earlier mobilization and shorter stay observed in the no-drain group.

Although this study was not designed as a comparative analysis of surgical techniques, the intrinsic technical attributes of the robotic platform likely played a role in the successful implementation of this no-drain protocol. The high-definition 3D visualization and multi-degree-of-freedom instruments facilitate precise hemostasis and intracorporeal suturing, ensuring a strictly dry field—a prerequisite for omitting the drain [24]. However, it is important to acknowledge that the technical advantages of the robotic platform do not eliminate biological realities. In our series, the decision to retain drains in 46.2% of cases was driven not by surgical failure but by physiological necessity. Specifically in myasthenia gravis cases requiring extensive mediastinal fat dissection, or in patients with dense pleuropulmonary adhesions, the resulting large raw surfaces create a risk of capillary oozing that even robotic precision cannot entirely nullify. In these instances, our protocol acted as a safety valve, ensuring that the “no-drain” goal never superseded the need for therapeutic drainage in complex anatomies.

### Limitations

Our study has several limitations. First, it is a single-center study with a relatively small sample size, which limits the power to detect rare complications. Second, although prospective, allocation was not randomized but determined by intraoperative safety criteria, which introduces confounding by indication: patients who received a drain inherently had specific intraoperative findings (air leak or oozing) that, although controlled, met the predefined exit criteria for drainage. The no-drain and drain groups therefore represent different intraoperative risk strata, and the observed differences in pain and length of stay should be interpreted as associations within a selected population rather than effects attributable to drain omission itself. Residual confounding related to operative time, tumor characteristics, and procedure type therefore cannot be excluded. The small cohort and protocol-driven treatment allocation preclude reliable adjusted (multivariable or propensity-score) estimates; therefore, only unadjusted comparisons are reported. In particular, the shorter stay in the no-drain group may be related to the absence of a chest tube but may also reflect differences in intraoperative complexity and postoperative course between the groups. Third, the cohort was heterogeneous in surgical procedure and pathology, with small numbers in each subgroup, so subgroup rates are descriptive and hypothesis-generating. Fourth, pain was assessed at a single early time point by an unblinded observer. Fifth, the “no-drain” decision relies on the surgeon’s assessment of hemostasis and air leak, although we standardized this process with the submerged air leak test to minimize subjectivity. Finally, although the reduction in hospital stay suggests economic benefits—consistent with the cost savings reported for enhanced recovery pathways in lung resection [25]—a formal cost-effectiveness analysis was not performed in this study.

## 5. Conclusions

In conclusion, this feasibility cohort suggests that omitting chest tubes in robotic mediastinal surgery is feasible and safe in appropriately selected patients who fulfill predefined intraoperative safety criteria, consistent with prior experience of chest tube omission after thoracoscopic and robotic mediastinal surgery [8,19] and with meta-analytic evidence supporting a no-drainage strategy after thoracoscopic resection [26]. In this cohort, the no-drain strategy was associated with lower early postoperative pain and discharge within the first 24 h, including in large and complex mediastinal lesions. These findings apply to carefully selected robotic mediastinal procedures performed at an experienced center and require multicenter prospective validation before broader adoption. Within this context, our data support a shift from routine drainage toward selective drainage, in which a chest tube is reserved for patients who meet predefined intraoperative criteria for drainage.

## Figures and Tables

**Table 1:** Baseline characteristics and demographics.

Variable	No-Drain Group (*n* = 21)	Drain Group (*n* = 18)	*p*-Value
Age (years), Mean ± SD	47.0 ± 12.8	43.0 ± 13.7	0.361
Gender (Male), *n* (%)	13 (61.9%)	10 (55.6%)	0.752
BMI (kg/m^2^), Mean ± SD	29.1 ± 5.1	27.8 ± 3.8	0.376
ASA Score, Median (IQR)	2.0 (2.0–2.0)	2.0 (1.0–2.8)	0.380
Smoking History, *n* (%)	11 (52.4%)	4 (22.2%)	0.098
Antiplatelet Use, *n* (%)	3 (14.3%)	2 (11.1%)	>0.99
Comorbidities, *n* (%)			
Hypertension (HT)	5 (23.8%)	4 (22.2%)	>0.99
Diabetes Mellitus (DM)	1 (4.8%)	2 (11.1%)	0.586
Myasthenia Gravis (MG)	3 (14.3%)	6 (33.3%)	0.255
Asthma	2 (9.5%)	2 (11.1%)	>0.99
Coronary Artery Disease (CAD)	1 (4.8%)	0 (0.0%)	>0.99
Chronic Kidney Disease (CKD)	0 (0.0%)	2 (11.1%)	0.206
COPD	0 (0.0%)	1 (5.6%)	0.462
Cerebrovascular Event (CVA)	1 (4.8%)	0 (0.0%)	>0.99
History of Malignancy	2 (9.5%)	0 (0.0%)	0.490
Tumor Size (mm), Median (IQR)	45.0 (30.0–76.0)	40.0 (4.6–55.0)	0.073

Abbreviations: *n*, number; SD, standard deviation; BMI, body mass index; ASA, American Society of Anesthesiologists physical status; IQR, interquartile range; HT, hypertension; DM, diabetes mellitus; MG, myasthenia gravis; CAD, coronary artery disease; CKD, chronic kidney disease; COPD, chronic obstructive pulmonary disease; CVA, cerebrovascular event.

**Table 2:** Intraoperative Parameters and Postoperative Outcomes.

Variable	No-Drain Group (*n* = 21)	Drain Group (*n* = 18)	*p*-Value
Intraoperative Parameters			
Total Operation Time (min), Median (IQR)	90.0 (65.0–185.0)	165.0 (120.0–203.8)	0.076
Console Time (min), Median (IQR)	75.0 (40.0–155.0)	125.0 (68.8–180.0)	0.067
Intraoperative Blood Loss (mL), Median (IQR)	0.0 (0.0–0.0)	0.0 (0.0–17.5)	0.458
Intraoperative Complications, *n* (%)	0 (0.0%)	1 (5.6%)	0.462
Conversion to Thoracotomy, *n* (%)	0 (0.0%)	1 (5.6%)	0.462
Postoperative Outcomes			
Chest Tube Duration (hours), Median (IQR)	-	24.0 (24.0–48.0)	N/A **
VAS Pain Score (Postop 12 h), Median (IQR)	2.0 (1.0–2.0)	5.0 (4.0–5.8)	<0.001 *
Length of Hospital Stay (hours), Median (IQR)	20.0 (18.0–36.0)	52.0 (39.0–56.0)	<0.001 *
Readmission within 30 days, *n* (%)	0 (0.0%)	1 (5.6%)	0.462
Postoperative Complications (Total), *n* (%)	2 (9.5%)	3 (16.7%)	0.647
Clavien–Dindo Grade I (Residual Pneumothorax)	2 (9.5%)	0 (0.0%)	
Clavien–Dindo Grade II (Wound Infection)	0 (0.0%)	1 (5.6%)	
Clavien–Dindo Grade IIIa (Pleural Effusion—Catheter)	0 (0.0%)	1 (5.6%)	
Clavien–Dindo Grade IIIb (Re-exploration—VATS)	0 (0.0%)	1 (5.6%)	
Reintervention (Catheter/Surgery)	0 (0.0%)	2 (11.1%)	0.206

* Statistically significant (*p* < 0.05). ** Chest tube duration was not compared between groups, as it is defined only in the drain group. Abbreviations: IQR, interquartile range; VAS, visual analog scale; postop, postoperative; VATS, video-assisted thoracoscopic surgery; N/A, not applicable.

**Table 3:** Stratification of drain usage by surgical procedure and pathological diagnosis.

Surgery Type and Pathology	Total (*n*)	No-Drain Group (*n*)	Drain Group (*n*)	No-Drain Rate (%)
Thymectomy	22	10	12	45.5%
Thymoma	7	5	2	71.4%
Thymic Cyst	10	4	6	40.0%
Thymic Hyperplasia	5	1	4	20.0%
Mediastinal Lesion Resection	17	11	6	64.7%
Mediastinal Cysts *	12	6	6	50.0%
Schwannoma	3	3	0	100.0%
Cystic Thymoma †	1	1	0	100.0%
Pulmonary Sequestration	1	1	0	100.0%
Overall	39	21	18	53.8%

* Includes bronchogenic (*n* = 5), mesothelial (*n* = 5), pericardial (*n* = 1), and esophageal (*n* = 1) cysts. † Cystic thymoma is a subtype of thymoma. This patient, operated on for a presumed thymic cyst, had a completion thymectomy after thymoma was confirmed; both procedures were drain-free. Thus, the eight thymoma procedures (no-drain rate 6/8, 75%) were performed in seven patients. Abbreviation: *n*, number of procedures.

## Data Availability

The data presented in this study are available on request from the corresponding author. The data are not publicly available due to privacy restrictions.

## Supplementary Materials

The following supporting information can be downloaded at: https://www.mdpi.com/article/10.3390/jcm15166438/s1, File S1: STROBE Statement—Checklist of items that should be included in reports of cohort studies.
