# Supplementary material for: Omitting Chest Tubes in Robotic Mediastinal Surgery: A Prospective, Protocol-Driven Analysis of Safety and Recovery
Source: J Clin Med. 2026 Aug 20;15(16):6438. doi: 10.3390/jcm15166438 (PMC13513774; doi:10.3390/jcm15166438)
Supplement: Supplementary file 1 [file jcm-15-06438-s001.zip › jcm-4450009-supplementary.pdf]

Supplementary Material

STROBE Statement — Checklist of items that should be included in reports of cohort studies

Manuscript: Omitting Chest Tubes in Robotic Mediastinal Surgery: A Prospective, Protocol-Driven Analysis of Safety and Recovery

No.	Item	STROBE Recommendation	Reported in (Section)
**Title and abstract**			
**1**	Title and abstract	(a) Indicate the study's design with a commonly used term in the title or the abstract	Title; Abstract (Methods) — "prospective" study; described as a prospective observational cohort in Methods 2.1
		(b) Provide in the abstract an informative and balanced summary of what was done and what was found	Abstract (structured: Background, Methods, Results, Conclusions)
**Introduction**			
**2**	Background/ rationale	Explain the scientific background and rationale for the investigation being reported	Section 1 (Introduction), paragraphs 1–3
**3**	Objectives	State specific objectives, including any prespecified hypotheses	Section 1 (Introduction), final paragraph (aim and hypothesis)
**Methods**			
**4**	Study design	Present key elements of study design early in the paper	Section 2.1 (single-center, prospective observational feasibility/protocol-implementation cohort)
**5**	Setting	Describe the setting, locations, and relevant dates, including periods of recruitment, exposure, follow-up, and data collection	Section 2.1 (single center; enrollment December 2024–December 2025); Section 2.5 (follow-up)
**6**	Participants	(a) Give the eligibility criteria, and the sources and methods of selection of participants; describe methods of follow-up	Section 2.1 (consecutive patients >18 y; inclusion/exclusion criteria); Section 2.5 (follow-up methods)
		(b) For matched studies, give matching criteria and number of exposed and unexposed	*Not applicable (not a matched study)*
**7**	Variables	Clearly define all outcomes, exposures, predictors, potential confounders, and effect modifiers. Give diagnostic criteria, if applicable	Section 2.5 (Primary efficacy endpoints and primary safety endpoint; Secondary endpoints); exposure = no-drain vs drain per Section 2.3
**8**	Data sources/ measurement	For each variable of interest, give sources of data and details of methods of assessment (measurement). Describe comparability of assessment methods if there is more than one group	Sections 2.5 (VAS by attending thoracic surgeon; chest radiographs; electronic medical record) and 2.6
**9**	Bias	Describe any efforts to address potential sources of bias	Section 2.1 and Section 4 (Limitations): confounding by indication explicitly addressed; groups defined as different intraoperative risk strata; results framed as associations
**10**	Study size	Explain how the study size was arrived at	Section 2.1 (feasibility/pilot cohort; no a priori sample-size calculation; consecutive eligible patients; not powered to exclude uncommon adverse events)
**11**	Quantitative variables	Explain how quantitative variables were handled in the analyses. If applicable, describe which groupings were chosen and why	Section 2.6 (normality testing; continuous variables as mean ± SD or median [IQR]); No-Drain vs Drain groups defined by protocol
**12**	Statistical methods	(a) Describe all statistical methods, including those used to control for confounding	Section 2.6 (Student's t-test, Mann–Whitney U, Fisher's exact/Fisher–

No.	Item	STROBE Recommendation	Reported in (Section)
			Freeman–Halton; Hodges–Lehmann median differences with 95% CIs; Clopper–Pearson 95% CIs)
		(b) Describe any methods used to examine subgroups and interactions	Sections 2.6 and 3.6 (pathology/procedure subgroups; exploratory bubble-chart visualization; no inferential regression modeling)
		(c) Explain how missing data were addressed	Section 3.1 (there were no missing data for the analyzed variables)
		(d) If applicable, explain how loss to follow-up was addressed	Section 2.5 (30-day follow-up complete for all patients, 38/38, 100%)
		(e) Describe any sensitivity analyses	*Not applicable (no sensitivity analyses performed)*
**Results**			
**13**	Participants	(a) Report numbers of individuals at each stage — e.g., numbers potentially eligible, examined for eligibility, confirmed eligible, included in the study, completing follow-up, and analysed	Section 3.1 (39 consecutive procedures in 38 patients; No-Drain n=21, Drain n=18; 100% follow-up)
		(b) Give reasons for non-participation at each stage	Section 2.1 / 3.1 (no eligible patients excluded; consecutive series)
		(c) Consider use of a flow diagram	Section 3.1 (participant flow described in text; consecutive series with no exclusions)
**14**	Descriptive data	(a) Give characteristics of study participants and information on exposures and potential confounders	Section 3.1 and Table 1 (demographics, comorbidities, tumor size)
		(b) Indicate number of participants with missing data for each variable of interest	Section 3.1 (no missing data for the analyzed variables)
		(c) Summarise follow-up time (e.g., average and total amount)	Section 2.5 (scheduled visits at ~12 and ~30 days; 30-day follow-up complete)
**15**	Outcome data	Report numbers of outcome events or summary measures over time	Section 3.4 and Table 2 (complications by Clavien–Dindo grade; primary safety endpoint 0/21); Section 3.3 (pain, length of stay)
**16**	Main results	(a) Give unadjusted estimates and, if applicable, confounder-adjusted estimates and their precision (e.g., 95% CI). Make clear which confounders were adjusted for and why they were included	Sections 3.1, 3.3, 3.4 (median differences with 95% CIs; no-drain rate 53.8% [95% CI 37.2–69.9%]; safety 0/21 [exact 95% CI 0–16.1%]). No adjusted models fitted (feasibility cohort)
		(b) Report category boundaries when continuous variables were categorized	Section 2.5 (VAS threshold ≤4 vs >4 for analgesia step-up; no-drain criteria)
		(c) If relevant, consider translating estimates of relative risk into absolute risk for a meaningful time period	Sections 3.1, 3.6 and Table 3 (absolute no-drain rates overall and by subgroup)
**17**	Other analyses	Report other analyses done — e.g., analyses of subgroups and interactions, and sensitivity analyses	Sections 3.5–3.6 and Table 3 (pathology/procedure stratification; exploratory visualizations, hypothesis-generating)
**Discussion**			
**18**	Key results	Summarise key results with reference to study	Section 4 (Discussion), first

No.	Item	STROBE Recommendation	Reported in (Section)
		objectives	paragraph
**19**	Limitations	Discuss limitations of the study, taking into account sources of potential bias or imprecision. Discuss both direction and magnitude of any potential bias	Section 4 (Limitations): single-center/small sample and power; confounding by indication; procedural heterogeneity; single unblinded pain assessment; surgeon-dependent decision; no cost-effectiveness analysis
**20**	Interpretation	Give a cautious overall interpretation of results considering objectives, limitations, multiplicity of analyses, results from similar studies, and other relevant evidence	Section 4 (Discussion): association-based interpretation in the context of prior literature
**21**	Generalisability	Discuss the generalisability (external validity) of the study results	Section 4 (Generalizability paragraph) and Section 5 (Conclusions): findings limited to appropriately selected patients at an experienced center; multicenter validation warranted
**Other information**			
**22**	Funding	Give the source of funding and the role of the funders for the present study and, if applicable, for the original study on which the present article is based	Funding statement (back matter): no specific grant received

Section references indicate the location of each item in the revised manuscript. Item numbering follows the STROBE Statement for cohort studies (von Elm E, et al. Lancet 2007;370:1453–1457). "N/A" denotes items not applicable to this single-center, prospective observational feasibility cohort.
